# Fertility disorders and pregnancy complications in hairdressers - a systematic review

**DOI:** 10.1186/1745-6673-5-24

**Published:** 2010-08-19

**Authors:** Claudia Peters, Melanie Harling, Madeleine Dulon, Anja Schablon, José Torres Costa, Albert Nienhaus

**Affiliations:** 1 Institute for Health Services Research in Dermatology and Nursing, University Medical Center Hamburg-Eppendorf, Martinistr. 52, 20246 Hamburg, Germany; 2 Institution for Statutory Accident Insurance and Prevention in the Health and Welfare Services, Department of Occupational Health Research Hamburg, Germany; 3 Occupational Health Division, Allergy and Clinical Immunology Division, Faculty of Medicine, Porto University, Porto, Portugal

## Abstract

**Background:**

Hairdressers often come into contact with various chemical substances which can be found in hair care products for washing, dyeing, bleaching, styling, spraying and perming. This exposure can impair health and may be present as skin and respiratory diseases. Effects on reproduction have long been discussed in the literature.

**Method:**

A systematic review has been prepared in which publications from 1990 to 2010 were considered in order to specifically investigate the effects on fertility and pregnancy. The results of the studies were summarised separately in accordance with the type of study and the examined events.

**Results:**

A total of 2 reviews and 26 original studies on fertility disorders and pregnancy complications in hairdressers were found in the relevant databases, as well as through hand searches of reference lists. Nineteen different outcomes concerning fertility and pregnancy are analysed in the 26 original studies. Most studies looked into malformation (n = 7), particularly orofacial cleft. Two of them found statistically significant increased risks compared to five that did not. Small for gestational age (SGA), low birth weight (LBW) and spontaneous abortions were frequently investigated but found different results. Taken together the studies are inconsistent, so that no clear statements on an association between the exposure as a hairdresser and the effect on reproduction are possible. The different authors describe increased risks of infertility, congenital malformations, SGA, LBW, cancer in childhood, as well as effects from single substances.

**Conclusion:**

On the basis of the identified epidemiological studies, fertility disorders and pregnancy complications in hairdressers cannot be excluded. Although the evidence for these risks is low, further studies on reproductive risks in hairdressers should be performed as there is a high public health interest.

## Background

People in many occupations are exposed to different requirements and stresses in their everyday working life. In hairdressing, women and men are exposed to physical and mental stress, as they have to stand for a long time. Moreover, they often come into contact with chemicals which are components in hair care products. The substances can be found in applications for dyeing or bleaching hair, for permanent waving and in styling products. The use of these preparations is an essential component of the occupation as hairdresser. Health can be impaired by the exposure to these chemicals. For example, widely used agents such as persulfates, organic solvents and endocrine disrupting chemicals were described in various reviews [1-3]. The chemicals may be absorbed by inhalation and/or through skin contact and may cause a variety of diseases, e.g. of the skin or the respiratory tract or even cancer [1,4]. Officially recognised occupational diseases of male and female hairdressers include skin diseases, as well as toxic irritant and allergic obstructive airway diseases [5-7]. There are numerous studies and publications on this topic in which different clinical pictures and triggering substances are described [8-10]. In contrast to this, the effects of occupational exposure on fertility and pregnancy of hairdressers and the foetal development of their children have more rarely been described, although there has long been some evidence that there might be unfavourable effects on reproduction [2].

For example, in 2007, 277,000 people were employed in the hairdressing occupation in Germany. Of these, 75% were 45 years old or younger. 90% of them were women; thus hairdressers are a significant group of employed women [11]. Once occupational health risks from exposure in the workplace have been recognised, actions may be possible to minimise the risk of unfavourable birth outcomes or of unwanted infertility.

This systematic review gives an overview of publications of epidemiological studies on the association between occupational exposure in the hairdressing profession and their effects on the fertility and pregnancy of women in this occupational group.

## Materials and methods

A search in MEDLINE from 1990-2010 was performed to search for epidemiological studies on health risks in hairdressers; the most recent update was performed on 31 May 2010. The key words refer to the occupational designations as well as to the different reproductive disorders. Diagnoses related to reproduction are especially unfavourable events, such as infertility and subfertility, prolonged time to pregnancy (TTP), spontaneous abortion, preterm birth, stillbirth, low birth weight (LBW < 2500g), small for gestational age (SGA), congenital malformations and delayed development of children. This led to the following search terms: *hairdresser/s, hair stylist/s, hairdressing occupation, hairdressing profession, professional hairdressing *or *professional hair care *in combination with *reproductive disorders, reproductive outcomes, fertility, infertility, subfertility, menstrual disorders, pregnancy outcomes, malformations, preterm birth, stillbirth, perinatal death, low birth weight/LBW, small for gestational age/SGA, spontaneous abortion*, as well as *time to pregnancy/TTP*.

Subsequently, further publications were identified from the lists of literature and used for the work.

The studies were selected in accordance with the following a priori defined inclusion criteria:

- Study design: Limitation to original studies - reviews and empirical studies

- Study content: With respect to the occupation as a risk factor and the occupational exposure

- Study population: Occupation as male or female hairdresser

- Outcome: Job-related health risks in reproduction

The studies are classified into occupational studies and hairdresser studies. Occupational studies examine multiple sections of the working population for a defined disease or exposure and differentiate the risks for different occupational groups. On the other hand, hairdresser studies define hairdressers as the study population and compare them with other populations or occupational groups with respect to health risks.

The most important characteristics and data of all included studies are shown in Tables 1 and 2. These present information on study design, place and time of the examination, as well as on the objective of the study and the tested population. Furthermore, the condition of the exposure and the results for the hairdressers are summarised. If single substances were mentioned as a possible cause of reproductive disorders in the hairdressing occupation, they are listed in the table. Descriptions of single publications can be found in the result section.

**Table 1:** Occupational studies: The risk of fertility disorders and pregnancy complications among hairdressers

Reference	Study design	Country, time	Study question	Study population	Exposure assessment	Result
Thulstrup 2006 [3]	Review	Medline, 1966-2004	Occupational exposures during pregnancy and birth defects	Employed women, 26 original studies	Occupation during pregnancy	Hairdressers No clear evidence for causal associations between maternal occupational exposures and specific birth defects

Garlantézec 2009 [15]	Cohort study	France, 2002-05	Risk of malformations and exposure to solvents	3,399 pregnant women (55 hairdressers) before 19^th ^gestational week	Self-reported exposure (never/occasional/regular, job exposure matrix (JEM) no/medium/high exposure	Hairdressers Self-reported: regular exposure JEM-assessed: medium exposure

Goulet 1991 [16]	Case-control study	Canada, 1982-84	Stillbirth and chemical exposure during pregnancy	227 stillbirths (> 20 weeks of gestation), 227 live births	Women working full-time (> 30 h/week) at the beginning of pregnancy, occupation, specific exposure to chemicals (light/moderate/high)	Hairdressers Odds Ratio (OR) 0.1* (#CI 0.0-0.3) P = 0.05

Kuijten 1992 [17]	Case-control study	USA, 1980-86	Childhood astrocytoma (< 15 years) and parental occupation	163 cases, 163 controls	Job categories, parental occupational history	Maternal occupation - hairdresser Preconception OR 2.5 (CI 0.4-26.2) Pregnancy OR 1.5 (CI 0.2-18) Postnatal OR 3.0 (CI 0.2-157.7)

Cordier 2001 [18]	Case-control study	Under IARC coordination in 7 countries, 1976-94	Childhood brain tumours and parental occupations	1,218 cases, 2,223 controls	Occupational history during 5-year period before child's birth	Maternal occupation - hairdresser OR 1.1 (CI 0.7-2.0)

Olshan 1999 [19]	Case-control study	Canada/USA, 1992-96	Childhood neuroblastoma (< 19 years) and parental occupation	538 cases, 504 controls	Occupational history, occupational groups	Maternal occupation - hairdresser OR 2.8 (CI 1.2-6.3) Paternal occupation - hairdresser OR 3.3 (OR 0.2-45.7)

Bianchi 1997 [12]	Case-control study	Italy, 1982-89	Congenital malformations and maternal occupation	1,791 cases, 3,223 controls	Maternal occupation during pregnancy	Hairdressers Orofacial clefts OR 2.2 (99% CI 0.4-10.7); Limb defects OR 2.2 (99% CI 0.8-6.1); multiple anomalies OR 1.7 (99% CI 0.7-4)

Lorente 2000 [13]	Case-control study	France/United Kingdom/Italy/Netherlands, 1989-92	Orofacial clefts and maternal occupational risk factors	100 cases (4 hairdressers), 751 controls (9 hairdressers)	Occupations before and during pregnancy, tasks, products handled, frequency of use	Hairdressers/beauticians Cleft palate only OR 5.1 (CI 1.01-25.9) Cleft lip with or without cleft palate OR 1.86 (CI 0.36-9.65)

Nguyen 2007 [20]	Case-control study	Norway, 1996-2001	Orofacial clefts and parental occupation	574 cases (without other malformations) (4 hairdressers), 763 controls (3 hairdressers)	Job title, industry type, status of work during early pregnancy (first 3 months)	Maternal occupation - hairdresser Cleft lip with or without palate OR 4.8* (CI 0.99-23) Cleft palate only OR 2.3* (CI 0.21-25)

Ormond 2009 [21]	Case-control study	England, 2000-03	Hypospadias and maternal occupational exposures to endocrine disrupting chemicals (EDCs)	471 cases, 490 controls	Job title, main tasks, self-reported exposure, job exposure matrix (JEM)	Hairdressers OR 2.59* (CI 0.7-12.3) Occupational exposure Hair spray OR 2.39*(CI 1.4-4.17) Phthalates OR 3.12*(CI 1.04-11.46)

Mutanen 2001 [22]	Register-based study	Sweden, 1958-96	Childhood cancer and parental occupation	8,185 cases < 15 years (45 hairdressers)	Job title	Hairdressers Kidney cancer (Father) Standardized Incidence Ratio (SIR) 10.6 (CI 2.9-27.2) (Mother) SIR 1.0 (CI 0.1-3.7)

Vrijheid 2003 [14]	Register-based study	England, Wales, 1980-96	Hypospadias and maternal occupational exposure to EDCs	3,471 cases (98 hairdressers), 35,962 controls -all congenital anomaly cases	Job title, exposure categories unlikely/possible/probable	Maternal occupation - hairdresser Observed/Expected Ratio (O/E) 0.99 (CI 0.81-1.19) (1980-89) O/E 0.94 (CI 0.74-1.17) (1992-96) O/E 1.18 (CI 0.8-1.64)

# The Confidence Interval (CI) is for the 95% level, unless otherwise noted.

* Adjusted for smoking

**Table 2:** Hairdresser studies: The risk of fertility disorders and pregnancy complications among hairdressers

Reference	Study design	Country, time	Study question	Study population	Exposure assessment	Results
Kersemaekers 1995 [2]	Review	Medline, 1985-1993	Reproductive disorders due to chemical exposure	Hairdressers; 9 studies	Hair washing, hair bleaching, hair dyeing, permanent waving, hair styling, solvents	Inconsistent results of studies, little evidence, reproductive risks cannot be excluded

Kersemaekers 1997 [25]	Cohort study	Netherlands, 1986-88, 1991-93	Reproductive disorders	4,236 hairdressers, 2,932 clothing sales clerks	Work at least 10 hours/week during the first 2 months of pregnancy	1st period: Spont. abortion OR 1.6 (#CI 1.0-2.4) Time to pregnancy (TTP) > 12 months OR 1.5 (CI 0.8-2.8) Low birth weight (LBW) OR 1.5 (CI 0.7-3.1) 2nd period: Abortion OR 0.9 (0.7-1.1)

Kersemaekers 1997 [26]	Cohort study	Netherlands, 1986-88, 1991-93	Neurodevelopment in offspring	4,236 hairdressers, 2,932 clothing sales clerks	Work at least 10 hours/week during the first 2 months of pregnancy	1st period: 1st word RR 2.4 (CI 1.1-5.1) 1st sentence RR 4,1 (CI 1.2-13.6) Seizures during fever RR 2.6 (CI 1.0-6.9) 2nd period: Decreased risks of seizures, no delayed child development

Rylander 2002 [27]	Cohort study	Sweden, 1973-94	Reproductive outcome	3,706 hairdressers, 3,462 reference population	Working time/week, treatments/week for permanent waving, hair dyeing, bleaching, shampooing, spraying	Small for gestational age (SGA) OR 1.4 (CI 1.1-1.7) Malformation OR 1.3 (CI 1.1-1.6) LBW OR 1.2 (CI 1.0-1.5) Preterm birth OR 1.1 (CI 0.9-1.3)

Zhu 2006 [28]	Cohort study	Denmark, 1997-2003	Pregnancy outcomes and developmental milestones children	550 hairdressers, 3,216 shop assistants	Working time/week, work postures	SGA OR 1.0*(CI 0.7-1.3) Preterm birth OR 1,0*(CI 0.7-1.6) Malformation OR 0.8*(CI 0.6-1.2) Fetal loss (spont. abortions + stillbirths) OR 0.7*(0.3-1.8) no differences in child development

Blatter 1993 [23]	Cross-sectional study	Netherlands, 1990	Menstrual disorders due to chemical exposure	64 hairdressers, 130 clothing shop assistants		Irregular cycle OR 2.4 (CI 1.1-5.2) Oligomenorrhoea OR 3.0 (CI 1.1-8.4) Unusual cycle length OR 3.4 (CI 1.5-7.8) Long blood loss OR 5.1 (CI 1.7-15.4) Severe pain OR 2.6 (CI 1.2-5.9)

Gan 2003 [29]	Cross-sectional study	China	Health effects due to exposure with permanent waving solution	57 hairdressers, 64 schoolteachers	Permanent waving procedure > 1 year	Menstrual disorders (menoxenia) Hairdressers 22.81% vs. reference 9.38% (p < 0.05)

Ronda 2009 [30]	Cross-sectional study	Spain, 2006	Menstrual disorders and subfertility	310 hairdressers, 310 shop assistants + office workers	No. of years in occupation, daily working hours previous year	Subfertility OR 2.17*(CI 0.91-5.17) Menstrual disorders OR 1.87*(CI 0.99-3.91)

Ronda 2010 [31]	Cross-sectional study	Spain, 2006	Pregnancy outcomes	310 hairdressers, 310 shop assistants + office workers	Job tasks, application of chemical products, ventilation	Spontaneous abortion OR 1.6*(0.9-2.7) Preterm birth OR 1.0*(0.4-2.9) LBW OR 0.2*(0.3-2.0)

Axmon 2006 [32]	Cross-sectional study	Sweden, 2000	Fertility/ time to pregnancy for wanted pregnancy and miscarriage risk	1,678 hairdressers, 1,578 referents population	Exposure before and during pregnancy, job tasks, ventilation	Fertility Fertility ratio (FR) 0.91 (CI 0.83-0.99) Spontaneous abortion OR 1.12 (CI 0.88-1.42)

Baste 2008 [33]	Cross-sectional study	Norway, 1997-99	Infertility, spontaneous abortion and smoking habits	136 hairdressers, 593 shop assistants/ 6,734 other occupations	Income for at least 100 hours in occupation last year	Infertility RR 1.3*(CI 1.08-1.55) Abortion RR 1.31*(CI 1.07-1.6) Hairdressers, never smoker: Infertility RR 2.01 (CI 1.45-2.8) Abortion RR 2.0 (CI 1.48-2.72)

Gallicchio 2009 [34]	Cross-sectional study	USA, 2005-08	Premature ovarian failure (POF)	443 hairdressers, 508 controls	Work history, employment status	POF RR 1.9*(CI 0.76-4.72) Caucasian women POF RR 3.24*(CI 1.06-9.91)

Rylander 2005 [35]	Register-based study	Sweden, 1983-2001	Reproductive outcome	8,384 hairdressers, reference: all deliveries 1983-2001 for working mothers	Working time during pregnancy (full-time/part-time)	SGA OR 1.19*(CI 1.07-1.33) LBW OR 1.10*(CI 0.99-1.21) Preterm birth OR 1.05*(CI 0.96-1.14)

Hougaard 2006 [36]	Register-based study	Denmark, 1998-2002	Risk of infertility	68 hairdressers, reference: all working women (20-44 years) + shop assistants	Economically active in registration	Infertility Relative risk (RR) 1.01 (CI 0.77-1.29) compared to shop assistants, RR 0.93 (CI 0.72-1.18) compared to all working women

Axmon 2009 [37]	Register-based study	Sweden, 1996	Comparison of birth weight and foetal growth	3,137 hairdressers and their sisters (3,952)	Graduates of vocational schools for hairdressers	Large for gestational age (LGA) OR 0.60*(CI 0.39-0.92), LBW OR 0.72*(CI 0.5-1.03), SGA OR 0.85*(CI 0.54-1.34)

Halliday-Bell 2009 [38]	Register-based study	Finland, 1990-2004	Adverse pregnancy outcome	10,622 hairdressers, 2,490 beauticians, 18,594 teachers	Working as a hairdresser	Hairdressers compared to teachers LBW OR 1.44*(CI 1.23-1.69) Preterm birth OR 1.21*(CI 1.07-1.38) SGA OR 1.65*(CI 1.38-2.07) Perinatal death OR 1.62*(CI 1.01-1.60)

# The Confidence Interval (CI) concerns the 95% level, unless otherwise noted.

* Adjusted for smoking

Additionally, the risk estimate for the target diagnosis of each study is presented in Table 3 and classified into statistically significant, not statistically significant deviation from one (risk estimate ≥ 1.5 or ≤ 0.5) and no association.

**Table 3:** Summary of epidemiological studies on the occupational risk of reproductive disorders among hairdressers

Outcome	Statistically significant	Non- significant*	No association	Reference
Infertility	+			Baste [33]
			±	Hougaard [36]

Subfertility		+		Ronda [30]
*Time to pregnancy*		+		Kersemaekers [25]

Menstrual disorders^§^	+			Blatter [23]
		+		Ronda [30]

Premature ovarian failure		+		Gallicchio [34]

Spontaneous abortion	1st period +		2nd period ±	Kersemaekers [25]
			±	Zhu [28]
			±	Ronda [31]
			±	Axmon [32]
	+			Baste [33]

Preterm birth			±	Rylander [27]
			±	Zhu [28]
			±	Ronda [31]
	+			Halliday-Bell [38]

Stillbirth	-			Goulet [16]
			±	Zhu [28]

Perinatal death	+			Halliday-Bell [38]

Small for gestational age	+			Rylander [27]
	+			Rylander [35]
			±	Zhu [28]
			±	Ronda [31]
			±	Axmon [37]
	+			Halliday-Bell [38]

Large for gestational age	-			Axmon [37]

Low birth weight		+		Kersemaekers [25]
	+			Rylander [27]
			±	Rylander [35]
			±	Axmon [37]
	+			Halliday-Bell [38]

Congenital malformation^§^		+		Bianchi [12]
	+			Rylander [27]
			±	Zhu [28]
*Hypospadias*				Vrijheid [14]
		+		Ormond [21]
*Orofacial cleft*		+		Bianchi [12]
		+		Nguyen [20]
*Cleft palate *				Lorente [13]
*Cleft lip*		+		Lorente [13]
			±	
				
				
	+			

Cancer				
*Kidney cancer*	Father +		Mother ±	Mutanen [22]
*Astrocytoma*		+		Kuijten [17]
*Neuroblastoma*	Mother +	Father +		Olshan [19]
*Brain tumour*			±	Cordier [18]

Delayed child	1st period +		2nd period ±	Kersemaekers [26]
development			±	Zhu [28]

**+ **Positive association

**- **Negative association

**± **No association

* Risk estimation ≥ 1.5 or ≤ 0.5

§ [15,29] not included because no effect estimate was given

## Results

The search query resulted in a total of 34 literature entries. 21 original studies from this were used for the review. The remaining studies did not fulfil the inclusion criteria and therefore could not be considered. Additionally, 7 relevant studies from the lists of literature in identified studies were used for the review. Therefore, a total of 28 publications on fertility disorders and pregnancy complications in hairdressers fulfilled the inclusion criteria. Mainly women were included in the examinations.

One review, as well as 11 original studies, concerns general occupational studies (Table 1) and on the other hand one review and 15 original studies refer to hairdresser studies (Table 2).

### 1. Occupational studies

Thulstrup et al. examined in their review the risks of specific birth defects in relation with maternal occupational exposure during pregnancy [3]. 26 studies, conducted between the years 1966 and 2004, were examined for the diagnoses: neural tube defects, orofacial clefts, congenital heart defects, urogenital abnormalities and limb defects. The authors found evidence for increased risks of a cleft lip or cleft palate in offspring of hairdressers. However, sufficient evidence for an association between congenital malformations and maternal occupational exposure during pregnancy was not found. Only 3 original studies reported findings for the occupational group of hairdressers [12-14]. These studies are included in the following section.

#### Cohort studies

A French prospective cohort study pursued the question of the risk of malformations due to exposure to solvents [15]. In this process, women before the 19th gestational week of pregnancy were included in the study and followed through birth. Exposure was estimated and assessed by using two different methods. On the one hand, self-provided information about the workplace was gathered and second, a Job Exposure Matrix (JEM) was used. In the JEM, the probability of solvent exposure for single occupations in comparison to the general population in different stages was given. Both methods showed significant exposure to organic solvents for hairdressers. According to the authors, associations were observed between orofacial clefts, urinary and male genital malformations in offspring and occupational exposure to solvents. Only a few hairdressers were included in the study (n = 55). According to the JEM, they were moderately exposed. The study does not show to what extent the children of hairdressers were affected by malformations.

#### Case control studies

A Canadian case control study dealt with the question of whether an occupational chemical exposure during pregnancy increases the risk of stillbirth [16]. The exposure of the mother was defined by her occupation and the contact with specific substances and classified as frequent/occasional/rare. A statistically significant reduction in the risk of stillbirths was found for infants born to hairdressers from the 20th gestational week. The authors assume that this result was correlated with other occupational or ergonomic factors.

In three American federal states, it was examined whether there is a link between the occupation of the parents and brain tumours in their offspring (astrocytoma) [17]. If the mother was a hairdresser before, during and after pregnancy, there was an increased risk of a childhood disease, though this was not statistically significant. Furthermore, the number of hairdressers was not given.

In contrast to this, in a study coordinated by the International Agency for Research on Cancer (IARC) in Europe, no association between brain tumours in children and the occupation of the parents as a hairdresser was found [18].

In another case control study, the risk of developing a neuroblastoma was examined in correlation with the occupation of the parents [19]. Children of hairdressers have an almost three-times higher and statistically significant risk of neuroblastoma in comparison to children whose mother has a different occupation. In contrast to this, there was no significant increase in the risk of this disease if the father was a hairdresser.

Bianchi et al. examined whether there was a possible association between the mother's occupation and teratogenic risks during pregnancy [12]. Increased risks of orofacial and multiple malformations, as well as limb anomalies, were found in the children of hairdressers. These risks were not statistically significant.

A multinational European study examined the risk of oral clefts in childhood in relation to the mother's workplace exposure [13]. Possible stress in the workplace was reflected through information on the occupation before and during pregnancy, particular occupational activities, the use of various products and the frequency of use. The result showed an increase of a factor of five and a statistically significant risk for the birth of a child with a cleft palate only for hairdressers and beauticians. The risk of a cleft lip with or without cleft palate was increased by a factor of almost two. However, this risk was not statistically significant. The proportion of hairdressers in the study population was very low, with a total of 13 (4 cases, 9 controls).

Nguyen et al. came to a similar result: they found that the children of hairdressers had an increased risk of the malformation orofacial clefts [20]. However, the proportion of hairdressers was also very low, with 4 cases and 3 controls.

In an English study, the occupational exposure of the mother to endocrine disrupting chemicals (EDCs) was examined with respect to hypospadias [21]. The result showed an increased risk for hairdressers as an occupational group. However, this risk was not statistically significant for this urogenital congenital anomaly of their male offspring. In comparison to non-exposed women, increased risks were also found for a self-reported occupational exposure to hair spray in the first trimester of pregnancy (OR 2.39; 95% CI 1.4 - 4.17). Contact with phthalates (used as a carrier substance in cosmetic products, such as hair sprays) showed a more than three-fold increase in the risk of hypospadias. On the other hand, folate supplementation showed evidence of a protective effect (OR 0.64, 95% CI 0.44-0.93).

#### Register-based studies

An increased incidence of renal cancer in the children of male hairdressers was found during an evaluation conducted by the Swedish Cancer Register on cancer in childhood and the occupation of the parents [22]. In contrast to this, no association between the occupation and cancer could be observed for female hairdressers.

Vrijheid et al. analysed data from the National Anomaly Register of Wales and England for the risk of hypospadias in correlation with the occupation of the mother and the exposure to potential endocrine disrupting chemicals [14]. All neonates recorded in the register from 1980 to 1996 were included as control group. The evaluation of this study showed no evidence for an increased risk of hypospadias in male offspring of female hairdressers. However, the authors pointed out that a large proportion of hairdressers are exposed to potential EDCs and particularly to phthalates. Other exposure to EDCs is possible. However, the exposure classification in this study is carried out only roughly, by using the occupational title.

In summary, 11 occupational studies were carried out in which hairdressers were included. Four of them showed a statistically significant risk of developing a reproductive disorder. However, these disorders are very different: an increase in risk was found for renal carcinoma if the father is a hairdresser [22], as well as for neuroblastoma [19] and cleft palate [13] if the mother is a hairdresser. Furthermore, 1 study showed a statistically significant reduction in the risk of stillbirths [16]. The risks posed by individual substances were not evaluated in the present studies. However, evidence for teratogenic effects when using solvents was presented [15]. An increased risk of hypospadias associated with maternal occupational exposure to phthalates in hair spray and other products suggest that EDCs may play a role in hypospadias [21].

### 2. Hairdresser studies

Kersemaekers et al. focused their review on the question of reproductive disorders in hairdressers through the exposure to chemicals [2]. In this process, the literature was examined for risks, such as infertility and subfertility, spontaneous abortions, congenital malformations, stillbirths, cancers in childhood and developmental disorders in association with hair care products. The composition of the products was evaluated for activities which are typical for this occupation and the reproductive effects of single substances were examined more closely. However, it was shown that the results of the present studies were inconsistent. Furthermore, risks during fertility and pregnancy in hairdressers cannot be excluded, however at the time of the review (1985-1993) only 2 studies with hairdressers [23,24] and 7 with all occupations were available, of which 2 studies fit within the determined time frame [16,17]. The numbered studies are described in this chapter.

#### Cohort studies

A retrospective cohort study performed in the Netherlands has analysed possible reproductive disorders in 4,236 hairdressers and 2,932 assistants in clothing shops [25]. The subjects worked for at least 10 hours per week during the first two months of pregnancy. The evaluation included possible changes in exposure through limitation of specific substances and a change in working conditions in the hair salon through improved ventilation and the use of gloves. In the first study period from 1986 to 1988, a statistically significant increase in the risk of spontaneous abortions for hairdressers was found. The risk could not be confirmed for the period from 1991 to 1993 or for the whole period of the study.

In another assessment of this cohort, Kersemaekers et al. compared the neurological development of children by using the age when they reached certain significant developmental phases: first step, first word and first sentence [26]. Furthermore, the occurrence of febrile convulsions as a possible indicator for abnormal neurological development was included in the analyses. In the first study phase, it was observed that language development in children of hairdressers was delayed. Furthermore, the seizures during fever were higher and the seizures were stronger the longer the mother worked during pregnancy. On the other hand, in the second study phase no delays in development were found and the risk of febrile seizures was also statistically increased only if the mother had worked until maternity leave (OR 1.8; 95% CI 1.1-3.2).

For the period from 1973 to 1994, a Swedish cohort study examined the reproductive outcomes of hairdressers with a control group from the general population [27]. In this process, the working time and specific hairdressing activities were considered with respect to the pregnancy outcomes. In comparison to women of the control group, an increased risk of SGA in infants born to hairdressers was found and their children were often affected by major congenital malformations. Associations of borderline statistical significance were observed for the effect of frequent shampooing and preterm births (OR 1.5, 95% CI 1.0-2.3) as well as for the weekly working time (> 30 hours) and LBW (OR 1.8, 95% CI 1.0-3.3).

Pregnancy outcomes among hairdressers and the developmental milestones of their children were examined in a Danish population-based prospective cohort study [28]. The exposure of the subjects was estimated on the basis of the weekly working time and on their work posture (standing, walking and standing, changing, sitting). Assistants in clothing shops were selected as a comparison group. There were no differences between the two groups with regard to fetal loss (spontaneous abortion and stillbirth), preterm birth, SGA and congenital malformation. No perinatal problems or delays in the development of the children during early childhood were observed.

#### Cross-sectional studies

In the Netherlands, Blatter et al. examined menstrual disorders through chemical exposure as an indicator for reproductive disorders [23]. In this process, the authors compared hairdressers with assistants in clothing shops as control groups. Only women between the ages of 20 and 45 years were included in the study. These women stated that they did not take any contraceptives. Due to this exclusion criterion and also due to the low response rate, the original chosen study population of 1,200 hairdressers and 1,200 controls was greatly reduced. Only the information on 64 hairdressers and 130 assistants in clothing shops could be used for the analysis. Nevertheless, an increased risk of menstrual disorders was observed for hairdressers. Statistically significant results were found for risks among hairdressers, such as irregular cycles, oligomenorrhoea, unusual cycle lengths, long blood loss, severe and protracted pain.

The aim of the study of Gan et al. was the examination of menstrual disorders in relation to permanent waving and exposure to the ingredient thioglycolic acid (TGA) [29]. They compared hairdressers with teachers and observed that menstrual disorders were significantly more frequent in exposed hairdressers than in the control group. The authors assume that reproduction in hairdressers is negatively influenced by long-term exposure to perm products due to the substance TGA.

In Spain, the effects of occupational activities on the reproductive health of hairdressers were examined in comparison to assistants in clothing shops and office employees [30]. The study focused on menstrual disorders such as short and long cycles, irregular cycles or missed period and intermenstrual bleeding, as well as subfertility, i.e. absence of pregnancy within 12 months of unprotected sexual intercourse. Chemicals in hair salons are assumed to be the cause for the increased but not statistically significant risks of menstrual disorders and subfertility.

In a further analysis of this data Ronda et al. showed results in pregnancy outcomes of hairdressers [31]. Increased risk was found for spontaneous abortion, mainly associated with work-related stress, but this result did not reach statistical significance. No differences between the two study groups for LBW and preterm birth were observed.

Axmon et al. examined whether occupational exposure as a hairdresser negatively influences fertility, as measured by the time until the beginning of pregnancy or the risk of spontaneous abortion [32]. In comparison to the general population, the time until the beginning of pregnancy was prolonged in hairdressers. On the other hand, no significant effect was observed with respect to abortions. Increased risks were found through the analysis of particular hairdressing activities and stressful working situations. However, these risks were not statistically significant.

The association between hairdressing and smoking and infertility and spontaneous abortion was examined in Norway [33]. The analysis was based on the data from a regional, cross-sectional study on cardiovascular diseases and their risk factors. Subjects who had worked less than 100 hours in the previous year were excluded. In comparison to other occupational groups, increased risks were found with respect to infertility and abortions after adjusting for smoking in hairdressers. Surprisingly, after adjusting for the smoking status, higher risks were observed for non-smoking hairdressers in comparison to their smoking colleagues.

In an American study on premature ovarian failure (POF), Gallicchio et al. compared the workplace exposure of hairdressers with other occupational groups [34]. The authors refer to animal experiments which assume an association between chemical exposure and POF. The diagnosis often results in infertility, as well as cardiovascular diseases and osteoporosis for women. A non-significant increase in risk was found for hairdressers in comparison to women of other occupational groups. The risk of POF for Caucasian hairdressers increased more than three-fold when the population groups were differentiated further. A relative risk of 5.58 (95% CI 1.24-25.22), adjusted for age and smoking, was observed for the age group between 40 and 55. Low response rates (21 or 35%) and the lack of a non-responder analysis are mentioned as limitations. As exposure assessments are not given, the results cannot be interpreted.

#### Register-based studies

Data from the Swedish Birth Register for the period 1983-2001 was used to examine the pregnancy outcomes of women who worked as hairdressers during pregnancy [35]. In this process, information on occupation and on working time (full/part-time) in the early pregnancy phase was recorded during the first visit to perinatal services. All other births during the period of the study were used as controls if the required information on the state of employment of the mother was available. An increased risk of small for gestational age of neonates was found in the group of all hairdressers, as well as in a subgroup of hairdressers who work full-time. However, the question of the cause could not be clarified as there was no information available in the birth register on occupation during the later phase of pregnancy or on specific exposure.

In Denmark, Hougaard et al. selected women with the diagnosis of infertility for a hospital-based study [36]. This diagnosis means that the subject did not become pregnant after at least 12 months of wanting a child. In Denmark, all women who were employed at the beginning of the study and between 20 and 44 years old were used as a comparison group and assistants in clothing shops were chosen as further controls. The result showed no difference for the number of hospital contacts due to infertility in hairdressers in comparison to the two control groups.

For a Swedish cohort of women who had graduated from vocational schools for hairdressers between 1970 and 1995 the parents were initially identified and subsequently the sisters through a register data search [37]. For the hairdressers and their sisters, information on pregnancy outcomes from the birth register was classified and then compared. The underlying hypothesis was that genetic factors or exposure during childhood might have significance in reproductive health. The result of the study showed a somewhat protective effect for hairdressers resulting in large for gestational age children (LGA). There were no risks of SGA and LBW associated with the occupation of the mother as hairdresser.

In Finland, the data from hairdressers, beauticians and teachers was selected from the National Birth Register and their risks of negative results of pregnancy were analysed in relation with the occupation [38]. As little or no occupational exposure was assumed for teachers, they were used as controls. The results were adjusted for possible confounders, such as age, marital status and smoking during pregnancy. In comparison to the teachers, statistically significant risks of LBW, preterm birth, SGA and perinatal death were found in neonates of hairdressers.

In summary, a statistically significant increase in risks to reproductive health, as well as a lack of association or a non-significant association can be shown (Table 3) for the hairdresser studies. With respect to fertility, significant risks of infertility [33] and menstrual disorders [23] were found. With respect to the pregnancy outcomes, 3 studies with increased risk of SGA [27,35,38], 2 of LBW [27,38] and 2 of spontaneous abortions [25,33] were available. However, Kersemaekers et al. observed this increased risk only in the early study phase [25]. Furthermore, statistically significant results were found for preterm birth [38], for perinatal death [38], for congenital malformations [27], a negative correlation for LGA [37], as well as a delay in childhood development in the early study phase [26].

## Discussion

This systematic review focuses on the effects of occupational exposure to chemical substances in hair care products on fertility and pregnancy in hairdressers. The review shows that the results of the included studies are inconsistent. No unambiguous association between the exposure in the workplace and the risk of reproductive disorders can be derived from the described studies. However, evidence for a possible increase in risks has been found repeatedly.

Studies on reproduction in hairdressers often target specific outcomes and the occurrence and frequency are compared with other population groups. The effects of specific substances on fertility and pregnancy in hairdressers are rarely the main focus.

Pregnancy outcomes were investigated in several studies with a wide range of different diagnoses. One of the most frequently examined outcomes in this context was the risk of spontaneous abortion among hairdressers. Increased risks were found in the study by Baste [33] and a Dutch cohort study, although this effect disappeared in the second study period [25]. Other studies did not identify any association between occupational exposure and spontaneous abortion [28,31,32]. An elevated risk of preterm birth in hairdressers was only found by Halliday-Bell [38]; other studies showed no differences between hairdressers and controls [27,28,31]. When considering deliveries of SGA newborns, a Swedish cohort study [27] and 2 register-based studies [35,38] found statistically significant effects among hairdressers. In contrast to this, the same number of studies did not find any association [28,31,37]. Similar findings were seen for the risk of LBW. A cohort study [27] and a register-based study [38] observed increased risks, and Kersemaekers et al. described a non-significant risk for hairdressers [25], although two studies did not confirm these results [35,37]. A further unfavourable pregnancy outcome is the occurrence of congenital malformations in offspring. Statistically significant associations were found for major malformations [27] and cleft palate only [13]; non-significant risks were observed for major malformations [12], hypospadias [21], orofacial clefts [12,20] and cleft lip [13]. No association was found between the risk of congenital malformations and the occupational exposure of hairdressers in 2 studies [14,28]. Malformations and childhood cancer were most often investigated in explorative studies, meaning that the studies related pregnancy outcomes to occupation in general and subsequently reported findings for hairdressers specifically. One of these studies found an increased risk of kidney cancer in the children of male hairdressers; no similar results were seen in the children of female hairdressers [22]. On the contrary, an association between neuroblastoma and maternal occupation were observed for hairdressers. No risk for the children of male hairdressers was found [19]. One study examined childhood astrocytoma and identified an increased although not significant risk for female hairdressers [17]. Brain tumours in children did not show any associations with the work environments of hairdressers [18]. Additional diagnoses were investigated more rarely. All results can be found in Table 3.

As shown in the review, the reports present varying results as regards reproductive disorders. Therefore, it is still possible that the health of hairdressers is at risk with respect to fertility and pregnancy.

Similar results were found in a review by Kersemaekers et al. 15 years ago [2]. To our knowledge, this is the only review to date that focused on reproductive disorders among hairdressers directly, even if they were only able to review a very small number of specific hairdresser studies. However, today additional studies on the reproductive risk of hairdressers are available, but inconsistent methods, outcomes and results make the drawing of conclusions difficult. There are several reasons why the studies are so inconsistent. First, the conception of reproductive disorders implicates a broad variety of adverse outcomes from infertility and other pre-pregnancy disorders to events during the perinatal or postnatal period through to child development. Second, a total of 26 original studies performed since 1990 were identified and only 15 studies are specific investigations on the exposure of hairdressers. The remaining studies are occupational studies and related pregnancy outcomes to occupation in general. Neither a comparison of the studies nor a final assessment is possible.

Thirdly, factors which explain the inconsistent results could include differences in treatments and fashion trends, differences in education and training between countries, changes in occupational environment, as well as different study designs and different reference populations.

Two different examination methods are used for risk assessment. The occupational studies focus on employees or specific exposure in general. With this study design, many occupational groups are examined, with the consequence that only a few hairdressers are included in the assessment. Small sample sizes lead to a low statistical power. This could be an explanation why in some studies elevated risks occurred in male or female hairdressers, but these were not statistically significant [12,20,21]. Based on the variety of occupations, the exposure can only be assessed very roughly, as a specific exposure is predominantly assigned to each occupational group and not as often on the basis of the actual work performed. However, such studies are important as they give evidence for possible health risks, which can be investigated through further studies.

In contrast to this, the hairdresser studies focus on the group of hairdressers and compare them with other occupational and/or population groups with respect to their occupational risk as regards reproduction. Assistants in clothing shops are preferred as a control group as they are considered to be rather similar with respect to the level of education, socioeconomic standing, and the physical and mental workloads. Additionally, different exposure in the workplace can be assumed, with the result that health risks for hairdressers can be observed as a factor of the activities typical of their occupation. Differences in age and smoking behaviour are often controlled by adjustment. With the exception of methodological deficiencies, this study design gives clear statements on hairdressers with respect to their risks. Many hairdressers participate in these studies and the exposure can be assessed more exactly.

Exposure comparable to that of the hairdressers can be assumed for the group of beauticians. In some countries hairdressers may also called cosmetologists, stylists or beauticians. Some studies have reported increases in the risks of spontaneous abortions [24], SGA [38] and low birth weights of children [39], as well as orofacial clefts [13]. Other studies did not find any association between occupational exposure among cosmetologists and menstrual disorders [40], infertility [41], congenital malformations [42], and other adverse pregnancy outcomes [43].

The exposure assessment is an essential problem in occupational studies. The assessment is used very differently in individual studies and ranges from the simple designation of the occupation "hairdresser" to the differentiated designation of the activity during which individual hair cosmetics are used, as well as their times of use. However, the occupation alone cannot serve as a substitute for data on exposure itself, as this does not permit its exact determination. Occupation only provides a rough measure of exposure, which can vary within the occupational group. Consequently, the assessment of the actual risks for hairdressers is very inaccurate and can possibly underestimate or overestimate the risks of hairdressing. A valid exposure assessment is necessary for an exact assessment of the occupational situation resulting in health risks. Hairdressing work can be associated with a variety of chemical contacts, which arise through the use of hair care products for washing, dyeing, bleaching, styling, spraying, as well as for perming, and media for cleaning and disinfecting the workplaces. In this environment, exposure is predominantly dermal or inhalative and depends in particular on the duration and frequency of the performed activities and their intensity. Precautions can minimise the exposure. If the room is adequately ventilated and the hairdresser wears gloves, the chemical exposure at the workplace can be reduced [44,45].

Another essential aspect is the study period. The working environment and also the hairdressing occupation are subject to constant change: older products are taken off the market and new formulations are used. Legal regulations and recommendations (e.g. for ventilation, for using gloves or for substituting or prohibiting certain ingredients) are changed due to new knowledge and thus can influence the exposure at the workplace and the health risks of hairdressers. Through the assessment of two time periods before and after changing the regulations in the Netherlands, the study of Kersemaekers et al. shows that the risks of pregnancy complications and developmental disorders was clearly reduced as a result of improved working conditions [25,26].

In addition to chemical exposure, the daily working time and physical stress, such as standing for a long period of time and unfavourable working postures, additionally influence the health of hairdressers. In a systematic review, Bonzini et al. showed that these factors increased the risk of preterm births and LBW children [46]. A meta-analysis confirmed associations between physically demanding work and preterm birth and SGA [47]. However, hairdressers were not considered in the studies.

## Conclusion

According to the available epidemiological studies, a risk of fertility disorders and pregnancy complications in hairdressers cannot be excluded. However, the evidence for increased reproductive disorders is currently low. Therefore, further studies on specific outcomes or on specific substances used in the hairdressing occupation and their adverse effects on reproductive health are needed.

## Competing interests

The authors declare that they have no competing interests.

## Authors' contributions

MH has made substantial contributions to the interpretation of data and has been involved in revising the manuscript critically for important intellectual content.

MD has made substantial contributions to the interpretation of data and has been involved in revising the manuscript critically for important intellectual content.

AS has made substantial contributions to the interpretation of data and has been involved in revising the manuscript critically for important intellectual content.

JTC has made substantial contributions to the interpretation of data and has been involved in revising the manuscript critically for important intellectual content.

AN has made substantial contributions to conception and design, the interpretation of data and has been involved in drafting and revising the manuscript critically for important intellectual content.

CP has made substantial contributions to conception and design, acquisition of data, the interpretation of data and has been involved in drafting and revising the manuscript.

All authors have read and approved the final manuscript.
